# Achievement emotions in AI-assisted learning, academic self-efficacy, and learning engagement and AI dependence among university students in statistics-related programs: a moderated mediation study

**DOI:** 10.3389/fpsyg.2026.1994136

**Published:** 2026-10-07

**Authors:** Huijuan Qin, Kun Liu

**Affiliations:** 1 College of Liberal Arts and Education, Hebei Open University, Shijiazhuang, Hebei, China; 2 College of Statistics and Mathematics, Hebei University of Economics and Business, Shijiazhuang, Hebei, China

**Keywords:** academic self-efficacy, achievement emotions, AI dependence, AI literacy, higher education, learning engagement, moderated mediation

## Abstract

**Background:**

Generative artificial intelligence (AI) tools have become part of everyday coursework for university students, yet the emotional and motivational conditions under which these tools support or undermine learning remain poorly understood. Drawing on the control-value theory of achievement emotions, this study examined whether academic self-efficacy mediates the links of positive achievement emotions in AI-assisted learning with learning engagement and AI dependence, and whether AI literacy moderates the first stage of these pathways.

**Methods:**

A cross-sectional survey was conducted with 700 undergraduate students (386 female; M_age = 19.81 years, SD = 1.27) majoring in applied statistics and related programs at three universities in Shijiazhuang, China. Participants completed measures of positive and negative achievement emotions, academic self-efficacy, learning engagement, AI dependence, and AI literacy. Confirmatory factor analysis preceded a first-stage moderated mediation analysis with bias-corrected bootstrap confidence intervals (5,000 resamples), controlling for negative emotions, gender, and year of study.

**Results:**

The six-factor measurement model fitted the data well, χ^2^(50) = 55.62, *p* = 0.271, CFI = 0.998, RMSEA = 0.013, SRMR = 0.017. Positive achievement emotions predicted higher self-efficacy (*β* = 0.374), which in turn predicted stronger engagement (*β* = 0.395) and lower AI dependence (*β* = −0.176), all *p* < 0.001, with AI literacy entered in every equation. AI literacy moderated the emotion-efficacy association (*β* = 0.152, *p* < 0.001): the conditional indirect association with engagement rose from 0.088 to 0.208 across ±1 SD of AI literacy, and that with AI dependence fell from −0.039 to −0.093. The direct association with AI dependence was positive (*β* = 0.447, *p* < 0.001), and the conditional total association remained positive (0.355 to 0.408). Both indexes of moderated mediation were significant.

**Conclusion:**

Positive emotional experiences in AI-assisted coursework show beneficial and costly associations simultaneously, and self-efficacy statistically accounts for opposing parts of each. The total association with dependence remained positive at all literacy levels, so the results describe inconsistent mediation rather than protection. AI literacy marks where the emotion-engagement pathway is strongest.

## 1 Introduction

Generative artificial intelligence tools have moved from technical curiosity to routine study infrastructure within a few years. Early reviews of AI applications in higher education anticipated a period of rapid, pedagogically under-theorized adoption, and that is what has occurred (Zawacki-Richter et al., 2019). Discussing the arrival of large language models, Kasneci et al. (2023) argued that these systems create genuine opportunities for personalized tutoring and feedback while demanding new competencies from learners. Acceptance research confirms that students perceive the tools as useful and intend to keep using them (Strzelecki, 2024). What remains far less settled is the psychological account of how AI-assisted studying connects to the quality of learning.

What the technology does to learning therefore cannot be separated from who is using it and how. The same AI session can scaffold a student’s reasoning or quietly replace it, and early commentaries stressed that outcomes would depend on learner competencies and instructional design rather than on the tool alone (Kasneci et al., 2023). This observation directs attention toward individual difference variables that shape the meaning AI tools acquire in a student’s academic life. Emotions and beliefs about competence are prime candidates, because both respond to the daily texture of AI-assisted study, its quick wins and its quiet frustrations.

On the outcome side, the evidence is genuinely mixed. A recent meta-analysis of experimental studies concluded that ChatGPT-based instruction improves academic performance relative to conventional conditions (Deng et al., 2025). At the same time, a systematic review of over-reliance on AI dialog systems reported that heavy, unreflective use is associated with weakened critical thinking, metacognition, and memory performance (Zhai et al., 2024). The cognitive offloading literature offers a mechanism for this pattern: when a tool reliably stores or computes information for us, the internal processes that would otherwise maintain those skills begin to decline through disuse (Risko and Gilbert, 2016). Survey evidence linking frequent AI tool use to lower critical-thinking performance through cognitive offloading extends the same logic to students (Gerlich, 2025). The same tool, then, can function as tutor or as crutch, and the field still lacks accounts of when each pattern emerges.

One way to organize these individual differences is motivational. Offloading research locates the risk of AI use in the displacement of internal cognitive work; engagement and efficacy research locates the benefit in the energy and confidence that productive tools can release. The two literatures have developed largely in parallel, and studies that model a beneficial and a costly outcome of AI-assisted learning within one design remain scarce (Zhai et al., 2024). A model that links the emotional experience of AI-assisted study to engagement and dependence simultaneously, through a shared motivational mechanism, would help connect them.

Achievement emotions are a natural starting point for such an account. Within the control-value theory of achievement emotions, emotions arise when learners appraise the control they hold over achievement activities and the value those activities carry, and these emotions in turn shape attention, motivation, and strategy use (Pekrun, 2006; Pekrun and Perry, 2014). Enjoyment and pride are positive activating emotions that widen attention and encourage persistence, whereas anxiety and boredom narrow or withdraw attentional resources (Pekrun et al., 2011). Most of this evidence comes from conventional classrooms. AI-assisted learning changes the appraisal context in specific ways: feedback arrives instantly, errors can be corrected without social exposure, and the learner confronts answers whose quality is often difficult to judge. How achievement emotions operate in this setting has only begun to be examined.

Two features of AI-assisted learning are especially relevant to control and value appraisals. Feedback from a language model is immediate and abundant, which should strengthen perceived control during practice; at the same time, the opacity of model outputs makes verification difficult for novice learners, which can undermine it. Error becomes socially costless, because a machine does not judge, yet effortful comprehension remains as demanding as ever. Whether these conditions strengthen or weaken the emotion-to-motivation sequence documented in classrooms may depend on the competencies students bring, which is where AI literacy enters the account.

The role of negative achievement emotions also requires attention. Anxiety and boredom are among the most commonly reported emotions in technology-mediated study, and they correlate with lower self-efficacy and maladaptive tool use (Pekrun et al., 2011). Because positive and negative emotions are moderately negatively correlated, an observed association between positive emotions and an outcome could reflect the absence of negative emotions instead. Entering negative emotions as covariates in every equation isolates the unique contribution of the positive side of the emotional profile, which is the focus of our hypotheses.

Academic self-efficacy has strong claims on any such account. Beliefs about personal competence determine how much effort students invest, how long they persist at difficult problems, and how they interpret failure (Bandura, 1997; Zimmerman, 2000). Students who trust their own capabilities approach complex material strategically rather than defensively, a pattern documented as early as Pintrich and De Groot (1990) work linking self-efficacy to self-regulated learning. Learning engagement captures the observable consequence of such beliefs: a persistent, positive state of vigor, dedication, and absorption in study (Schaufeli et al., 2002a). Engagement predicts attainment and adjustment in university samples (Schaufeli et al., 2002b) and is among the most widely used indicators of learning quality in educational research (Fredricks et al., 2004). Positive achievement emotions should feed engagement both directly and through the confidence students place in their own competence.

One ordering decision requires explicit justification. Within control-value theory, control appraisals and competence beliefs are typically treated as antecedents of achievement emotions, and reciprocal effects of emotions on appraisals are assumed over time (Pekrun, 2006). We model the emotion-to-efficacy direction for two reasons. First, affective states are a recognized source of efficacy information: students read their emotional reactions during an activity as evidence about their capabilities (Bandura, 1997). Second, the emotions examined here are experienced during AI-assisted study itself, and the proximal question of this study is what these in-activity emotions are associated with, rather than how control beliefs generate them. We fitted the reverse-direction first stage as a sensitivity check; it explained a similar share of variance (R^2^ = 0.278 vs. 0.258), so the cross-sectional data cannot adjudicate the ordering, and we return to this point in the Limitations.

Engagement has also become a standard outcome in research on AI in higher education. Studies report that AI tool use relates to engagement through motivational mediators such as autonomous motivation (Ahmed and Sultan, 2026), and that technostress from AI use can suppress engagement, with AI literacy buffering the effect (Su, 2026). What these studies leave open is the antecedent side: whether the feelings students develop while working with AI tools reach engagement only through belief in their own competence. That question matters practically, because emotions are more malleable than many cognitive traits, and interventions that reshape the emotional climate of coursework are within reach of ordinary instructors.

AI dependence describes the other side of the coin: a pattern in which students lose confidence in completing academic tasks unaided and default to AI tools even when the task does not warrant them (Zhai et al., 2024). Recent work shows that dependence is already measurable in Chinese universities, where perceived usefulness of generative AI was associated with generative AI dependence in a large student sample (Liu and Liu, 2026). Dependence is not simply high usage. A student who deploys an AI tool deliberately to overcome a bottleneck is using a resource; a student who cannot start an assignment without one has outsourced a competence. Whether AI-assisted learning tips toward engagement or dependence may depend on who is using the tool. AI literacy, the set of competencies needed to understand, evaluate, and work with AI systems (Ng et al., 2021), can now be measured at the individual level with validated instruments (Wang et al., 2023). Literate users can verify outputs, recognize failure modes, and keep the cognitive work of learning for themselves, which suggests that AI literacy conditions how emotional experiences of AI-assisted study relate to self-belief. Three recent studies position this configuration of variables squarely within current scholarship: differential AI effects on self-efficacy and intrinsic motivation (Shao and Wang, 2026), motivational mediation of AI effects on engagement (Ahmed and Sultan, 2026), and AI literacy as a moderator of technostress effects on engagement (Su, 2026).

Taken together, prior work documents that AI-assisted learning can raise performance, that over-reliance carries cognitive costs, that emotions and self-efficacy drive engagement in conventional settings, and that AI literacy conditions how students handle AI tools. What is missing is an integrated account testing whether the emotional experience of AI-assisted coursework is linked to both a beneficial and a costly outcome through academic self-efficacy, and whether AI literacy gates those links. The contribution we aim for is substantive rather than methodological: not moderated mediation as a technique, but evidence on when positive emotional experiences with AI tools accompany confidence and engagement rather than reliance on the technology. The present study addresses this gap in a sample of applied statistics undergraduates, a population for whom AI assistants have become part of everyday quantitative work.

The present study integrates these strands in a single moderated mediation model. We asked two questions about the emotional experience of AI-assisted learning among applied statistics undergraduates. First, do positive achievement emotions in AI-assisted coursework predict learning engagement and AI dependence through academic self-efficacy? Second, does AI literacy moderate the emotional construction of self-efficacy, and thereby the strength of the indirect pathways? Statistics coursework is a useful test bed because it combines procedural practice, conceptual difficulty, and heavy current reliance on computational tools, including AI assistants for code and interpretation. One prediction deserves advance justification. Positive emotions toward AI-assisted work should also attract students to the tools themselves: activities that feel good get repeated, and frequent, affectively rewarding AI use is a plausible gateway condition for dependence (Zhai et al., 2024). We therefore expected the direct association of positive emotions with AI dependence to be positive, while the pathway through self-efficacy was expected to be negative. Five hypotheses guided the analysis. We expected that positive achievement emotions would positively predict learning engagement (H1); that academic self-efficacy would mediate the relation between positive achievement emotions and engagement (H2); that positive achievement emotions would show a positive direct association with AI dependence while the indirect path through self-efficacy would be negative (H3); that AI literacy would moderate the association between positive emotions and self-efficacy, with a stronger association at higher literacy (H4); and that the indirect effects of positive emotions on both outcomes through self-efficacy would be moderated by AI literacy (H5).

## 2 Materials and methods

### 2.1 Participants and procedure

Participants were 700 undergraduate students recruited from applied statistics and related programs (Applied Statistics, *n* = 329; Statistics, *n* = 204; Data Science and Big Data Technology, *n* = 167) at three universities in Shijiazhuang, Hebei Province, China. All participants were at least 18 years old (M = 19.81, SD = 1.27, range 18.0 to 23.8). The sample included 386 women (55.1%) and 314 men (44.9%); 351 students (50.1%) reported a rural household registration. Year of study was distributed as follows: first year, *n* = 206; second year, *n* = 199; third year, *n* = 168; fourth year or above, *n* = 127. Self-reported frequency of AI tool use for coursework spanned the full response range, with 41 students reporting never or almost never, 103 rarely, 226 sometimes, 216 often, and 114 always or almost always.

The three participating universities were selected to span institutional profiles: a provincial key university, a regular undergraduate institution, and an applied-technology college, all located in Shijiazhuang. Recruitment targeted intact class groups in statistics and data science programs, where AI tools are commonly used for programming assistance, output interpretation, and report drafting. In total, 24 classes participated (9, 8, and 7 classes at the three universities; mean class size 29.2 students, range 27 to 31). The study focused on applied statistics education because statistical work combines conceptual judgment with procedural execution, giving AI tools a role in both.

Data were collected between March and May 2026. During regular class sessions, instructors distributed a QR code linking to an anonymous online questionnaire hosted on the Wenjuanxing platform. The first page presented the study purpose, the voluntary nature of participation, and the informed consent statement; proceeding to the items constituted consent. All participants were adults and provided their own consent. No course credit or payment was offered. Of 782 students invited, 739 returned questionnaires (94.5%). We excluded 31 protocols showing invariant responding (the same response option on at least 95% of items) and 8 protocols with more than 10% missing values, leaving a final sample of 700 (89.5% of invited students). Simulation-based sensitivity analyses (1,000 replications) indicated power of approximately 0.80 for detecting first-stage interaction effects of |β| = 0.09 and power above 0.98 for the index of moderated mediation at the magnitudes observed here; the sample also provides power above 0.90 for detecting population correlations of 0.13 or larger (two-tailed, α = 0.05). The research was approved by the Ethics Committee of Hebei Open University and conducted in accordance with the Declaration of Helsinki.

### 2.2 Measures

All items used a five-point Likert scale ranging from 1 (strongly disagree) to 5 (strongly agree). Scale wording was adapted to refer specifically to AI-assisted study of statistics, following the recommendation that context-specific adaptation improves validity (Pekrun et al., 2011). Item scores were averaged within scales, with higher values indicating higher levels of the construct.

All instruments were administered in Chinese. Where source items were in English, they were translated and then independently back-translated by two bilingual graduate students, with discrepancies resolved through discussion. Adapted items kept the original response format but replaced the object of each statement with reference to AI tools used in statistics coursework, such as chat assistants, code generators, and automated explainers.

Positive achievement emotions were measured with eight items adapted from the Achievement Emotions Questionnaire (Pekrun et al., 2011), four for enjoyment (α = 0.88; e.g., “I enjoy working through statistical problems with the help of AI tools”) and four for pride (α = 0.88; e.g., “I feel proud when I solve a difficult exercise after checking the AI explanation”). The two facets correlated strongly and were combined into a total score (α = 0.91). Enjoyment and pride are both positive, activating emotions that share control and value antecedents in control-value theory (Pekrun, 2006), which justifies aggregation; sensitivity analyses with each emotion modeled separately are reported in the Results.

Negative achievement emotions were measured with eight items from the same source, four for anxiety (α = 0.85; e.g., “I feel tense when the output an AI tool gives me does not make sense”) and four for boredom (α = 0.84; e.g., “Checking AI-generated answers bores me”). The total score (α = 0.89) served as a covariate in all models; sensitivity analyses entering anxiety and boredom as separate covariates are reported in the Results.

Academic self-efficacy was measured with eight items adapted from the self-efficacy subscale of the Motivated Strategies for Learning Questionnaires (Pintrich and De Groot, 1990) to the AI-assisted statistics context (e.g., “I am confident I can understand the statistical concepts of this course even when AI explanations are imperfect”). Facet reliabilities were 0.84 and 0.83, and the scale reliability was 0.88. For the parcel-level measurement model, the eight items were assigned to two artificial parcels (Self-efficacy A and B) by alternating items ranked on item-total correlation; these parcels have no substantive meaning, and a single-factor item-level model for this scale is reported in the Results.

Learning engagement was measured with nine items from the student version of the Utrecht Work Engagement Scale (Schaufeli et al., 2002b), covering vigor (α = 0.85), dedication (α = 0.84), and absorption (α = 0.85; e.g., “Time flies when I am working on my statistics coursework”). The total score showed α = 0.91.

AI dependence was measured with seven items written for this study, covering two content domains identified in the over-reliance literature (Zhai et al., 2024) and in recent measurement work on generative AI dependency (Liu and Liu, 2026): difficulty completing academic tasks without AI (four items) and uncritical acceptance of AI output (three items). The items are: “I find it hard to finish my assignments without AI tools”; “When an AI tool is unavailable, I do not know how to start my statistics homework”; “I need AI tools to check my work before I feel confident that it is correct”; “Completing a statistical analysis task entirely on my own feels almost impossible”; “When an AI tool gives an answer, I rarely question it”; “I usually use AI-generated content after only a quick look”; and “If an AI tool’s output looks reasonable, I see no need to verify it against other sources.” An initial pool of nine items was reviewed by two statistics instructors and one educational researcher for content coverage and wording; two items overlapping with general usage frequency were removed, so that the scale targets dependence rather than frequency of use or favorable attitudes. Item-level evidence for the two-domain structure is reported in the Results. The scale reliability was α = 0.90.

AI literacy was measured with eight items adapted from the Artificial Intelligence Literacy Scale (Wang et al., 2023), retaining two content areas of the AI literacy framework (Ng et al., 2021): understanding of AI (four items: “I understand how the AI tools I use for statistics coursework generate their answers”; “I know the strengths and limitations of AI tools for statistical analysis”; “I can tell which statistics tasks AI tools are suited for and which they are not”; “I understand why an AI tool sometimes produces incorrect or fabricated output”) and use and evaluation of AI (four items: “I can judge whether an answer produced by an AI tool is trustworthy”; “I can verify AI-generated statistical results using other methods”; “I can write clear prompts to obtain the analysis I need from AI tools”; “I can detect and correct errors in AI-generated code or explanations”). Item selection prioritized the competencies most relevant to verifying AI output in statistics learning; the ethical and affective dimensions of the original framework were not retained, and this narrowing is considered in the Limitations. Item-level evidence for the two-area structure is reported in the Results. The scale reliability was α = 0.92.

### 2.3 Data analysis

Analyses were performed in Python 3.13 using the pandas, statsmodels, and semopy libraries. Scale reliabilities were estimated with Cronbach’s alpha and McDonald’s omega, the latter from item-level factor models (Hayes and Coutts, 2020). Because all measures were self-reported, common method variance was addressed procedurally (anonymity, confidentiality assurances, and varied item wording) and examined statistically in three ways: Harman’s single-factor test, for which the first unrotated factor accounted for 23.5% of total variance; full collinearity variance inflation factors, all below 1.71 and thus under the 3.3 criterion (Kock, 2015); and a latent method factor model, in which all substantive loadings remained substantial (0.63 to 0.92). Harman’s test alone cannot establish the absence of method bias (Podsakoff et al., 2003), and we treat these results as convergent but not definitive evidence. The measurement model was tested with confirmatory factor analysis (maximum likelihood estimation) on 13 facet-level parcels (two per construct, three for engagement), with fit judged against the criteria of CFI and TLI at or above 0.95, RMSEA at or below 0.06, and SRMR at or below 0.08 (Hu and Bentler, 1999). Because several instruments were shortened or adapted to the AI-assisted statistics context, parcel-level results were supplemented with item-level factor analyses for every construct and with heterotrait-monotrait ratios (HTMT) for discriminant validity (Henseler et al., 2015). Convergent validity was evaluated through average variance extracted, with values of 0.50 or higher considered acceptable, and discriminant validity by comparing the square root of each construct’s average variance extracted with its correlations with the other constructs (Fornell and Larcker, 1981). Intraclass correlations were trivial at the university level (all at or below 0.005) and at the class level (absolute values at or below 0.014 across the 24 classes), with design effects of approximately 1.0; as a safeguard, all focal regressions were additionally estimated with class-cluster-robust standard errors.

Before model estimation, item-level distributions were screened. Skewness ranged from −0.27 to 0.15 across the 48 items, and no scale showed floor or ceiling concentration. After screening, no item-level missing values remained, so no imputation was required. Facet scores served as indicators in the confirmatory factor analysis because parceling reduces idiosyncratic item noise while preserving the theoretical structure: enjoyment and pride represented positive emotions, anxiety and boredom represented negative emotions, two balanced artificial parcels represented self-efficacy, the two content domains represented AI dependence and AI literacy respectively, and the three engagement dimensions represented the engagement factor. We acknowledge that parceling can conceal item-level misfit; the item-level analyses reported below address this risk. Standardized coefficients are reported throughout; the interaction term was constructed from standardized predictors, and its coefficient represents the change in the slope of positive achievement emotions per one standard deviation increase in AI literacy.

The moderated mediation hypotheses were tested with a first-stage moderated mediation design (Preacher et al., 2007; Hayes, 2018). Academic self-efficacy was regressed on positive achievement emotions, AI literacy, their interaction, and the covariates; each outcome was regressed on academic self-efficacy, positive achievement emotions, AI literacy, and the same covariates. AI literacy was entered in the outcome equations because it correlated with both engagement and AI dependence, and omitting it could bias the estimated associations of self-efficacy with the outcomes. Negative achievement emotions, gender, and year of study entered every equation as covariates, with gender coded 1 = female and 0 = male. All continuous predictors were standardized before the product term was formed. Regression coefficients are ordinary least-squares estimates, and the reported z statistics are coefficient-to-standard-error ratios. Indirect effects were estimated at three levels of AI literacy (one standard deviation below the mean, the mean, and one standard deviation above the mean), with bias-corrected bootstrap confidence intervals based on 5,000 resamples (Preacher and Hayes, 2008). Conditional total effects, the sum of the direct effect and the conditional indirect effect at each level of AI literacy, are reported alongside the conditional indirect effects, and the total associations of positive emotions with both outcomes, estimated without the mediator, are reported to evaluate the hypotheses at the total-effect level. Moderated mediation was assessed with the index of moderated mediation, the slope of the indirect effect as a function of the moderator.

## 3 Results

### 3.1 Preliminary analyses

Table 1 reports descriptive statistics, reliabilities, and correlations. All six scales showed good internal consistency (α between 0.88 and 0.92). Positive achievement emotions correlated moderately with academic self-efficacy (*r* = 0.44), learning engagement (*r* = 0.42), and AI literacy (*r* = 0.30), and weakly with AI dependence (*r* = 0.20). Negative achievement emotions showed the expected negative associations with self-efficacy (*r* = −0.29) and engagement (*r* = −0.25) and a positive association with AI dependence (*r* = 0.19). Self-efficacy correlated strongly with engagement (*r* = 0.52) and weakly and negatively with AI dependence (*r* = −0.12). AI literacy correlated negatively with AI dependence (*r* = −0.26). The correlation patterns were consistent with the hypothesized model.

**Table 1:** Descriptive statistics, reliabilities, and bivariate correlations (*N* = 700).

Variable	M	SD	α	1	2	3	4	5
1. Positive achievement emotions	3.54	0.66	0.91	—				
2. Negative achievement emotions	3.11	0.72	0.89	−0.31***	—			
3. Academic self-efficacy	3.50	0.58	0.88	0.44***	−0.29***	—		
4. Learning engagement	3.44	0.64	0.91	0.42***	−0.25***	0.52***	—	
5. AI dependence	2.82	0.78	0.90	0.20***	0.19***	−0.12**	−0.08*	—
6. AI literacy	3.30	0.61	0.92	0.30***	−0.13***	0.22***	0.27***	−0.26***

Scales scored 1 to 5. *p < 0.05, **p < 0.01, ***p < 0.001.

At the facet level, means ranged from 2.78 to 3.66, with enjoyment highest and the dependence facets lowest, indicating that students on average experienced moderately positive emotions in AI-assisted study while reporting dependence slightly below the scale midpoint. Gender differences were uniformly small across the six scales (Cohen’s d between −0.15 and 0.14), as were associations with year of study (r between −0.08 and 0.06), supporting their use as covariates rather than grouping factors.

### 3.2 Measurement model

The six-factor model fitted the data well, χ^2^(50) = 55.62, *p* = 0.271, CFI = 0.998, TLI = 0.998, RMSEA = 0.013 (90% CI [0.000, 0.028]), SRMR = 0.017. Standardized loadings of the 13 facet parcels ranged from 0.78 to 0.84, all *p* < 0.001 (Table 2). Average variance extracted was 0.66 for positive achievement emotions, 0.67 for negative achievement emotions, 0.65 for academic self-efficacy, 0.65 for learning engagement, 0.66 for AI dependence, and 0.66 for AI literacy. The square roots of these values (0.81 to 0.82) exceeded every inter-construct correlation, supporting discriminant validity (Fornell and Larcker, 1981).

**Table 2:** Standardized loadings and convergent validity from the confirmatory factor analysis.

Construct	Facet parcel	Loading
Positive achievement emotions	Enjoyment	0.82
	Pride	0.80
Negative achievement emotions	Anxiety	0.84
	Boredom	0.79
Academic self-efficacy	Self-efficacy A	0.79
	Self-efficacy B	0.82
Learning engagement	Vigor	0.84
	Dedication	0.79
	Absorption	0.78
AI dependence	Dependence A	0.82
	Dependence B	0.80
AI literacy	AI literacy A	0.82
	AI literacy B	0.81
Average variance extracted	Positive emotions	0.66
	Negative emotions	0.67
	Self-efficacy	0.65
	Engagement	0.65
	AI dependence	0.66
	AI literacy	0.66

*N* = 700. All loadings significant at *p* < 0.001. Model fit: χ^2^(50) = 55.62, *p* = 0.271, CFI = 0.998, TLI = 0.998, RMSEA = 0.013 (90% CI [0.000, 0.028]), SRMR = 0.017. Square roots of average variance extracted (0.81 to 0.82) exceeded all inter-construct correlations.

Item-level analyses largely supported the measurement structure beyond the parcel model, with one exception (Table 3). Facet-structured models fitted well for positive emotions, χ^2^(19) = 16.64, *p* = 0.614 (enjoyment and pride factors), negative emotions, χ^2^(19) = 13.42, *p* = 0.816 (anxiety and boredom), and engagement, χ^2^(24) = 29.87, *p* = 0.189 (vigor, dedication, and absorption factors). For AI dependence, a two-factor item-level model separating difficulty without AI from uncritical acceptance fitted the data well, χ^2^(13) = 9.28, *p* = 0.751, CFI = 1.00, RMSEA < 0.001, with all loadings between 0.79 and 0.85; the two factors correlated at 0.75, supporting the use of a total score while acknowledging the two content domains. For AI literacy, a two-factor model separating understanding of AI from use and evaluation also fitted well, χ^2^(19) = 12.86, *p* = 0.845, CFI = 1.00, RMSEA < 0.001, with loadings between 0.81 and 0.85 and a factor correlation of 0.74. A single-factor model for the eight self-efficacy items did not meet the stated fit criteria, χ^2^(20) = 225.56, *p* < 0.001, CFI = 0.916, RMSEA = 0.121, and we therefore no longer describe this fit as acceptable. All eight standardized loadings remained salient (0.67 to 0.73) and McDonald’s omega was 0.88, so the misfit reflects local rather than global measurement failure. Inspection of standardized residual correlations showed modest local dependencies (largest absolute value 0.10) concentrated among four of the items, consistent with item-level wording overlap. Two alternative specifications clarify the source of the misfit. A one-factor model with the corresponding residual covariances freed remained below the criteria, χ^2^(16) = 156.89, CFI = 0.943, RMSEA = 0.112, indicating that the dependencies are diffuse rather than attributable to a single item pair. A two-factor model separating the two item clusters fitted the data well, χ^2^(19) = 14.95, *p* = 0.726, CFI = 1.00, RMSEA < 0.001, with loadings between 0.73 and 0.78 and a factor correlation of 0.775, supporting essential unidimensionality with local item dependencies. We retained the balanced parcels in the main measurement model for three reasons: the two-factor solution indicates that the misfit reflects local dependencies rather than a violation of unidimensionality; a cross-cluster reassignment of the items to new parcels also fitted well, χ^2^(50) = 74.44, *p* = 0.014, CFI = 0.994, RMSEA = 0.026; and the focal regressions were estimated on composite scores rather than parcel scores, so they are mathematically unaffected by the parcel assignment, with all focal coefficients identical to the third decimal under the alternative assignment. McDonald’s omega ranged from 0.88 to 0.92 across the six scales, and all HTMT ratios were at or below 0.58, under the 0.85 criterion (Henseler et al., 2015).

**Table 3:** Item-level measurement models and McDonald’s omega (*N* = 700).

Construct	Item-level model	χ^2^(df)	*p*	CFI	RMSEA	ω
Positive emotions	Two factors (enjoyment, pride)	16.64(19)	0.614	1.00	<0.001	0.91
Negative emotions	Two factors (anxiety, boredom)	13.42(19)	0.816	1.00	<0.001	0.89
Academic self-efficacy	Single factor (suboptimal; see text)	225.56(20)	<0.001	0.916	0.121	0.88
Academic self-efficacy	Two factors (item clusters)	14.95(19)	0.726	1.00	<0.001	0.88
Learning engagement	Three factors (vigor, dedication, absorption)	29.87(24)	0.189	1.00	0.019	0.91
AI dependence	Two factors (content domains)	9.28(13)	0.751	1.00	<0.001	0.90
AI literacy	Two factors (content areas)	12.86(19)	0.845	1.00	<0.001	0.92

Maximum likelihood estimation. CFI values of 1.00 reflect fit values marginally above 1 rounded down. Factor correlations were 0.75 (AI dependence) and 0.74 (AI literacy). Heterotrait-monotrait ratios ranged from 0.09 to 0.58, all below the 0.85 criterion (Henseler et al., 2015). For academic self-efficacy, the single-factor item-level model did not meet the stated fit criteria; Section 3.2 reports the residual diagnosis and alternative specifications, and the two factors correlated at 0.775.

### 3.3 Moderated mediation analysis

Table 4 presents the regression results. In the self-efficacy equation, positive achievement emotions (a₁ = 0.374, z = 10.43, *p* < 0.001), AI literacy (a₂ = 0.102, z = 2.95, *p* = 0.003), and their interaction (a₃ = 0.152, z = 4.80, *p* < 0.001) were all significant predictors, and the equation explained 25.8% of the variance in academic self-efficacy. The significant interaction indicates that positive emotions were associated with self-efficacy more strongly among students with higher AI literacy: the conditional slope of positive emotions rose from 0.222 at one standard deviation below the mean of AI literacy to 0.374 at the mean and 0.526 at one standard deviation above the mean.

**Table 4:** Regression results for the moderated mediation model.

Equation	Predictor	β	SE	z	*p*
Academic self-efficacy (R^2^ = 0.258)	Positive achievement emotions (a₁)	0.374	0.036	10.43	<0.001
	AI literacy (a₂)	0.102	0.034	2.95	0.003
	Positive emotions × AI literacy (a₃)	0.152	0.032	4.80	<0.001
	Negative emotions	−0.165	0.035	−4.77	<0.001
	Female	0.154	0.066	2.32	0.020
	Year of study	0.025	0.033	0.75	0.452
Learning engagement (R^2^ = 0.335)	Academic self-efficacy (b₁)	0.395	0.035	11.16	<0.001
	Positive achievement emotions (c′₁)	0.193	0.036	5.33	<0.001
	AI literacy	0.112	0.033	3.42	<0.001
	Negative emotions	−0.065	0.033	−1.95	0.051
	Female	−0.114	0.063	−1.81	0.071
	Year of study	0.011	0.031	0.35	0.727
AI dependence (R^2^ = 0.241)	Academic self-efficacy (b₂)	−0.176	0.038	−4.66	<0.001
	Positive achievement emotions (c′₂)	0.447	0.039	11.56	<0.001
	AI literacy	−0.327	0.035	−9.33	<0.001
	Negative emotions	0.237	0.035	6.67	<0.001
	Female	0.011	0.067	0.17	0.867
	Year of study	−0.028	0.033	−0.83	0.404

*N =* 700. All continuous predictors standardized; gender coded 1 = female, 0 = male, and its coefficient is the female–male difference in outcome standard-deviation units. Interaction terms based on standardized predictors.

In the engagement equation, academic self-efficacy predicted engagement (b₁ = 0.395, z = 11.16, *p* < 0.001), positive achievement emotions retained a significant direct association (c′₁ = 0.193, z = 5.33, *p* < 0.001), and AI literacy contributed independently (*β* = 0.112, z = 3.42, *p* < 0.001); the equation explained 33.5% of the variance. In the AI dependence equation, academic self-efficacy predicted lower dependence (b₂ = −0.176, z = −4.66, *p* < 0.001), positive achievement emotions showed a positive direct association (c′₂ = 0.447, z = 11.56, *p* < 0.001), and AI literacy predicted lower dependence (*β* = −0.327, z = −9.33, *p* < 0.001); the equation explained 24.1% of the variance. Among the covariates, negative achievement emotions predicted lower self-efficacy (*β* = −0.165, *p* < 0.001) and higher AI dependence (*β* = 0.237, *p* < 0.001). Gender showed a small association with self-efficacy (*β* = 0.154, *p* = 0.020) but not with the outcomes once AI literacy was entered; year of study was negligible in every equation (all |β| ≤ 0.03).

Conditional indirect effects appear in Table 5. At low AI literacy, the indirect effect of positive emotions on engagement through self-efficacy was 0.088 (95% CI [0.048, 0.134]); at the mean it was 0.148 (95% CI [0.112, 0.186]), and at high AI literacy it reached 0.208 (95% CI [0.160, 0.261]). The corresponding indirect effects on AI dependence were −0.039 (95% CI [−0.069, −0.019]), −0.066 (95% CI [−0.098, −0.038]), and −0.093 (95% CI [−0.137, −0.055]). Because no confidence interval included zero, all conditional indirect effects were significant. The index of moderated mediation was significant for engagement (0.060, 95% CI [0.035, 0.089]) and for AI dependence (−0.027, 95% CI [−0.045, −0.013]), confirming that AI literacy moderated both indirect pathways. The total association of positive emotions with engagement, estimated without the mediator, was 0.337 (z = 9.15, *p* < 0.001), so H1 holds at the total-effect level as well; the total association with AI dependence was 0.383 (z = 10.44, *p* < 0.001). Conditional total effects on AI dependence were 0.408 (95% CI [0.335, 0.480]), 0.382 (95% CI [0.308, 0.449]), and 0.355 (95% CI [0.280, 0.424]) at low, mean, and high AI literacy: the combined association remained positive across the observed range, and the self-efficacy pathway offsets, but does not reverse, the positive direct association. Hypotheses H1 through H5 were supported, with H3 interpreted as an inconsistent (competitive) mediation pattern rather than as evidence that positive emotions reduce AI dependence.

**Table 5:** Conditional indirect effects, conditional total effects, and index of moderated mediation (bootstrap = 5,000).

AI literacy level	Engagement	95% CI	AI dependence	95% CI
−1 SD (indirect)	0.088	[0.048, 0.134]	−0.039	[−0.069, −0.019]
Mean (indirect)	0.148	[0.112, 0.186]	−0.066	[−0.098, −0.038]
+1 SD (indirect)	0.208	[0.160, 0.261]	−0.093	[−0.137, −0.055]
Index of moderated mediation	0.060	[0.035, 0.089]	−0.027	[−0.045, −0.013]
−1 SD (total)	0.281	[0.201, 0.362]	0.408	[0.335, 0.480]
Mean (total)	0.341	[0.263, 0.417]	0.382	[0.308, 0.449]
+1 SD (total)	0.401	[0.316, 0.481]	0.355	[0.280, 0.424]

*N =* 700. Indirect and total effects of positive achievement emotions through academic self-efficacy at three levels of AI literacy; bias-corrected bootstrap confidence intervals with 5,000 resamples. Total effects are the sum of the direct effect and the conditional indirect effect.

In descriptive terms, the indirect pathway from positive emotions to engagement roughly doubled in strength across the observed range of AI literacy, while the negative indirect pathway involving AI dependence more than doubled. The pattern indicates that AI literacy does not redirect the motivational sequence but scales it.

Because participants were recruited through 24 intact classes, all focal regressions were re-estimated with class-cluster-robust standard errors. Class-level intraclass correlations were negligible (absolute values at or below 0.014 for the six composites), and every focal coefficient retained its sign and significance: the interaction of positive emotions with AI literacy (z = 3.96, *p* < 0.001), self-efficacy predicting engagement (z = 10.82, *p* < 0.001), self-efficacy predicting AI dependence (z = −6.36, *p* < 0.001), and the AI literacy coefficients in both outcome equations (z = 3.75 and z = −10.97, *p* < 0.001). The single-level specification is therefore not responsible for the observed pattern.

Simulation-based sensitivity analyses aligned with the moderated mediation design indicated power of approximately 0.80 for first-stage interaction effects of |β| = 0.09 (1,000 replications), well below the observed interaction of 0.152 (estimated power above 0.99), and Monte Carlo power above 0.98 for both indexes of moderated mediation at the observed magnitudes.

Sensitivity analyses also examined the emotion aggregates. With enjoyment or pride entered separately as the predictor, the pattern replicated: each emotion predicted self-efficacy (*β* = 0.347 and 0.311, both *p* < 0.001), each interaction with AI literacy was significant (*β* = 0.155 and 0.120, both *p* < 0.001), and the signs of all focal paths were unchanged. Entering anxiety and boredom as separate covariates likewise left every focal coefficient within 0.01 of the reported values. The findings therefore do not depend on the aggregation strategy.

## 4 Discussion

This study asked how the emotions students experience in AI-assisted coursework relate to their engagement and their dependence on AI tools, and whether AI literacy conditions these associations. Three findings stand out. First, positive achievement emotions were associated with both outcomes through academic self-efficacy, but in opposite directions: the indirect path ran toward stronger engagement and toward weaker AI dependence. Second, AI literacy moderated the association between positive emotions and self-efficacy, so that both indirect pathways were stronger as literacy increased. Third, positive emotions retained a positive direct association with AI dependence, and their conditional total association with dependence remained positive at all levels of AI literacy: the pattern is an inconsistent mediation, in which a smaller indirect pathway pulls against a larger positive combined association.

Each hypothesis found support. Positive achievement emotions were associated with engagement at the total-effect level (0.337) as well as directly and through self-efficacy (H1, H2). The direct association with AI dependence was positive while the indirect pathway through self-efficacy was negative (H3), a pattern best described as inconsistent mediation. AI literacy moderated the first stage, with a stronger emotion-efficacy association at higher literacy (H4), and moderated both indirect effects, as the significant indexes of moderated mediation confirm (H5). The coherence of the full pattern with the theoretical account strengthens the interpretation, although the cross-sectional design keeps it provisional.

Read through control-value theory, the pattern is coherent. AI-assisted study supplies conditions under which enjoyment and pride arise readily: rapid feedback, visible progress, and repeated moments of success (Pekrun, 2006). The emotional experience of those moments marks motivational differences, and where those differences appear depends on what the student can do with the tool. Among literate students, positive emotions were linked to stronger beliefs about personal competence, which in turn accompanied engaged study. Among students with weaker AI literacy, the same positive emotions showed markedly weaker associations with self-efficacy, and correspondingly weaker indirect links to both outcomes.

### 4.1 Theoretical implications

The first contribution is substantive rather than methodological: documenting the simultaneous adaptive and maladaptive correlates of positive emotional experiences in AI-assisted learning, and identifying the conditions under which the adaptive side is strongest. The study also extends control-value theory into AI-assisted learning. The theory holds that emotions reflect appraisals of control and value and feed back into attention, motivation, and strategy use (Pekrun, 2006; Pekrun and Perry, 2014). Our results show that this motivational sequence still operates when part of the cognitive work is delegated to a tool. Students who enjoyed and took pride in AI-assisted statistics study believed more in their own competence, and that belief was in turn associated with energetic, absorbed engagement, the state that the UWES tradition measures (Schaufeli et al., 2002a; Fredricks et al., 2004). The negative indirect association with AI dependence adds a motivational pathway that engagement research rarely considers: confidence in one’s own competence appears to mark a boundary on outsourcing. The pattern is consistent with the reading that students with high self-efficacy can use AI tools without depending on them, which is precisely the difference between resource use and dependence.

Treating engagement and dependence as simultaneous outcomes also carries a measurement lesson. Engagement scales ask about energy and absorption in study generally, and dependence scales ask about the felt necessity of AI tools; the two were nearly orthogonal in this sample (*r* = −0.08), yet both are fed by the same antecedent through opposite pathways. Studies that model only one of the two will mischaracterize the net effect of emotional and motivational variables on AI-assisted learning. Designs that include both outcomes offer a more honest account of what AI tools are doing to students.

The moderation result gives AI literacy a specific theoretical role. Rather than acting as a direct antidote to dependence, literacy amplified the association between positive emotions and self-efficacy. One reading is that literacy determines what a student can actually do with an emotionally positive disposition. Enjoyment motivates exploration, but only a learner who can verify outputs, debug code, and recognize erroneous answers can turn that exploration into mastery experiences, the raw material of self-efficacy (Bandura, 1997; Zimmerman, 2000). For students low in AI literacy, positive feelings about AI-assisted study may coexist with passive consumption of outputs, a pattern consistent with accounts of cognitive offloading (Risko and Gilbert, 2016) and with survey evidence linking offloading to weaker critical thinking (Gerlich, 2025).

The moderation also refines how control appraisals should be understood in AI settings. Control in conventional classrooms derives from skill and effort; in AI-assisted study, control is distributed between the learner and the tool. A student with high AI literacy retains judgment over the division of labor, deciding what to delegate and what to verify, which preserves the subjective sense of control that both enjoyment and self-efficacy require (Pekrun and Perry, 2014). A student with low literacy faces outputs that arrive faster than they can be evaluated, so the experience of success attaches to the tool rather than the self. The amplified emotion-to-efficacy pathway at high literacy is consistent with this reading.

The positive direct association between positive emotions and AI dependence (c′₂ = 0.447), alongside the negative indirect pathway, deserves careful interpretation. The conditional total association remained positive at low (0.408), mean (0.382), and high (0.355) AI literacy, so the data give no support to the claim that positive emotional experiences reduce dependence overall; what self-efficacy provides is a countervailing pathway that weakens, without reversing, the positive combined association. This inconsistent mediation pattern parallels the coexistence of benefits and risks documented in the experimental literature (Deng et al., 2025) and in reviews of AI over-reliance (Zhai et al., 2024), and it aligns with recent evidence that AI effects on motivational and engagement outcomes are conditional rather than uniform (Shao and Wang, 2026; Ahmed and Sultan, 2026; Su, 2026). Enjoying AI-assisted work evidently accompanies heavier reliance on these tools, and our design cannot separate fondness for the tools from fondness for the work. What the model adds is the qualification that the self-efficacy route runs in the opposite direction, toward autonomy, and that AI dependence can be modeled as an outcome alongside engagement, following recent measurement work in Chinese samples (Liu and Liu, 2026).

### 4.2 Practical implications

For statistics educators, three implications follow, each stated as a hypothesis for future intervention research rather than as a prescription established by the present cross-sectional evidence. First, the emotional texture of AI-assisted coursework may matter pedagogically. Task designs that preserve opportunities for success experiences, recognize correct reasoning rather than correct answers alone, and keep challenge within the range where control appraisals form (Pekrun, 2006) are candidates for supporting the emotion-efficacy-engagement sequence observed here, but their effects need experimental tests. Second, AI literacy may be a condition for turning AI use into learning. Embedding verification practice, prompt evaluation, and error detection into the curriculum builds the competencies (Ng et al., 2021; Wang et al., 2023) that our moderation result identifies as the amplifier of the emotional pathway; whether such training changes the moderated pattern is an open intervention question. Third, dependence deserves monitoring in its own right. An instructor who sees students unable to begin assignments without AI assistance is seeing the behavioral signature of outsourced competence; assessment formats that require unaided reasoning on some components, followed by AI-supported extension on others, are a plausible safeguard (Zhai et al., 2024).

A further implication concerns sequencing, again as a hypothesis. Because the emotion-efficacy association was strongest among literate students, literacy training that precedes or accompanies emotionally positive AI-assisted tasks might yield larger motivational returns than literacy training that follows them. In applied statistics programs, this argues for testing early verification habits: checking model assumptions an assistant omits, reproducing a generated analysis by hand, and comparing answers across tools. Whether such exercises turn the instant feedback that AI provides into genuine mastery experiences (Bandura, 1997) is a question for designed interventions.

### 4.3 Limitations and future directions

Several limitations qualify these conclusions. The cross-sectional design cannot establish causal order, and the direction we modeled is not the only defensible one: control-value theory typically treats competence beliefs as antecedents of emotions, and our reverse-direction sensitivity check explained a similar share of variance (R^2^ = 0.278 vs. 0.258), so the data cannot adjudicate the ordering; longitudinal or experimental work is needed. All measures were self-reported, and although the statistical checks (Harman’s test, full collinearity VIFs, and a latent method factor model) did not indicate pervasive method bias, common method variance cannot be excluded. The AI dependence scale was developed for this study and, although its two-domain structure fitted well and reliability was high, further validation with test–retest data, behavioral criteria, and independent samples is needed; the AI literacy measure was shortened to two content areas and does not cover the ethical and affective dimensions of the original framework. The main measurement model relied on facet parcels, which can conceal item-level misfit; we mitigated this risk with item-level analyses for every construct, but the two strategies answer slightly different questions. In particular, the one-factor item-level model for academic self-efficacy showed suboptimal fit owing to local dependencies among items; although a two-factor solution fitted well and the focal results do not depend on the parcel assignment, the item-level structure of this adapted scale requires further examination in independent samples. The sample came from applied statistics and related programs at three universities in one Chinese city, and students in statistical disciplines may differ from the general undergraduate population in quantitative preparation and tool usage; the findings should not be generalized to other disciplines or educational levels without further evidence. Self-reported AI use frequency, rather than logged usage, underlies our description of the sample. Future studies should include behavioral traces of AI use, metacognitive calibration measures, designs that can separate attraction to AI tools from genuine engagement, and intervention tests of the practical hypotheses outlined above.

## 5 Conclusion

Among Chinese undergraduates in statistics-related programs at three universities in one city, positive achievement emotions in AI-assisted coursework were associated with stronger learning engagement and, through academic self-efficacy, with lower AI dependence, and AI literacy moderated both indirect pathways. The direct and total associations with AI dependence remained positive, however, so the results describe an inconsistent mediation pattern rather than a protective effect of positive emotions. Within these cross-sectional and disciplinary boundaries, the findings identify academic self-efficacy as a motivational correlate that statistically distinguishes the engagement-linked side of positive emotional experiences from the dependence-linked side, and AI literacy as the condition under which the emotion-efficacy association is strongest. Longitudinal and intervention designs are needed to test whether these associations can be turned into causal leverage.

## Data Availability

The raw data supporting the conclusions of this article will be made available by the authors, without undue reservation.
